# Evaluation of pitolisant, sodium oxybate, solriamfetol, and modafinil for the management of narcolepsy: a retrospective analysis of the FAERS database

**DOI:** 10.3389/fphar.2024.1415918

**Published:** 2024-11-11

**Authors:** Xiaodan Zhou, Jia Chen, Bangtian Xu, Li Chen

**Affiliations:** ^1^ Department of Pharmacy, University-Town Hospital of Chongqing Medical University, Chongqing, China; ^2^ Department of Pharmacy, Chengdu Jinniu District People’s Hospital, Chengdu, China; ^3^ Department of Pharmacology, University of the Basque Country UPV/EHU, Leioa, Spain; ^4^ Department of Pharmacy and Evidence-Based Pharmacy Center, West China Second University Hospital, Sichuan University, Chengdu, China; ^5^ Key Laboratory of Birth Defects and Related Diseases of Women and Children, Ministry of Education, Sichuan University, Chengdu, China

**Keywords:** narcolepsy, pitolisant, sodium oxybate, solriamfetol, modafinil, FAERS

## Abstract

**Objective:**

Narcolepsy, a rare neurological disorder believed to have an autoimmune etiology, necessitates lifelong management. This study aimed to provide evidence supporting the safety of pharmacological treatment for narcolepsy.

**Methods:**

Five-year data on pitolisant, sodium oxybate, solriamfetol, and modafinil were extracted from the FDA Adverse Event Reporting System (FAERS) self-reporting database for the period spanning from 2019 to 2023. Various statistical methods, including the reporting odds ratio (ROR), proportional reporting ratio (PRR), Bayesian confidence propagation neural network analysis (BCPNN), and multi-item gamma Poisson shrinker (MGPS), were employed to quantify the signals. Finally, a comparative analysis was conducted between demographic data, outcomes, and inherent associations among the medications and the signals.

**Results:**

After data analysis, we obtained 50 signals (a cumulative count of 762 cases) for pitolisant, 640 signals (corresponding to 46,962 cases) for sodium oxybate, 40 signals (equivalent to 1,228 cases) for solriamfetol, and finally, 72 signals (representing 632 cases) for modafinil. The majority of these patients were female. Psychiatric and nervous system disorders were identified as the predominant adverse drug events (ADEs). For sodium oxybate, it is crucial to consider psychiatric disorders (such as suicidal ideation), respiratory disorders (including sleep apnea syndrome and respiratory depression), and signs of pregnancy and congenital familial diseases. For solriamfetol, noteworthy new ADEs include drug inefficacy, suicidal ideation, restless legs syndrome, and somnambulism. Furthermore, a relationship has been observed between modafinil use and restricted fetal growth, spontaneous abortion, cognitive disorders, and drug inefficacy and abuse.

**Conclusion:**

The majority of observed adverse reactions in this study were consistent with those listed in the product instructions. However, potential novel or notable ADE signals were identified through real-world pharmacovigilance analysis. It is anticipated that this paper will offer additional information regarding safe and rational medication for narcolepsy.

## 1 Introduction

### 1.1 Background

Narcolepsy is a rare neurological disorder that typically necessitates lifelong treatment. Global prevalence estimates indicate that approximately 0.02%–0.05% of individuals are affected (Ohayon et al., 2002; Partinen and Kronholm, 2017). The onset of narcoleptic symptoms usually occurs at an early age and significantly impacts the academic performance and daily life of patients during their school years and personality development period (Scammell, 2015). Narcolepsy primarily presents with a range of sleep-wake cycles and other symptoms. The main clinical manifestations include excessive daytime sleepiness (EDS), cataplexy, hypnagogic hallucination, sleep paralysis and nocturnal sleep disturbance (Thorpy and Krieger, 2014). EDS is often the most troublesome feature, contributing to a decline in patients’ quality of life (Bassetti et al., 2019). Currently available treatments for narcolepsy are symptomatic, targeting sleepiness, cataplexy and disrupted nocturnal sleep (Humphreys et al., 2024).

Pitolisant, sodium oxybate (SO), solriamfetol and modafinil are commonly recommended medications for managing narcolepsy, especially EDS (Bassetti et al., 2021). Pitolisant is a histamine-3 (H3) receptor antagonist/inverse agonist indicated for the treatment of EDS or cataplexy in patients with narcolepsy (Dauvilliers et al., 2013). SO is the sodium salt of gamma-hydroxybutyrate (GHB), an endogenous compound and metabolite of the neurotransmitter gamma aminobutyric acid (GABA) (Schneider et al., 2023). Solriamfetol is a dopamine and norepinephrine reuptake inhibitor (DNRI) indicated to improve wakefulness (Yang and Gao, 2019). Modafinil can improve EDS symptoms by 65%–90% (Morgenthaler et al., 2007). Although these drugs are recommended as first-line treatments, concerns still exist regarding their adverse effects. Some patients frequently discontinue or switch to alternative medications due to drug ineffectiveness or serious adverse reactions. Given the relatively small number of narcolepsy patients, the evidence supporting its safety compared to other drugs is relatively weak. Therefore, conducting a pharmacovigilance study based on an adverse event database is particularly crucial.

### 1.2 Objectives

The FDA Adverse Event Reporting System (FAERS) serves as a pivotal resource for the retrospective analysis of real-world adverse drug events (ADEs) (Shu et al., 2022; Tian et al., 2022). The present investigation is dedicated to harnessing the FAERS database for the extraction and analysis of ADE-related signals, with the ultimate goal of augmenting the safety profile of narcolepsy therapeutics. This is achieved by examining the disproportional signals associated with pitolisant, sodium oxybate (SO), solriamfetol, and modafinil, which are recognized therapeutic agents for narcolepsy. By identifying and evaluating these signals, this study aimed to illuminate the comparative safety profiles of the aforementioned drugs, thereby informing clinical decisions and enhancing the efficacy and safety of narcolepsy management.

## 2 Methods

### 2.1 Data sources

The FAERS database is a spontaneous reporting system that relies on voluntary reports and quarterly updates (U.S. Department of Health and Human Services, 2024). The data mining process will be conducted from 2019 to 2023, utilizing seven types of datasets (DEMO, DRUG, REAC, OUTC, RPSR, THER and INDI). MySQL is employed for the management of FAERS data. In total, 7,552,239 ADE reports were documented after deleting duplicate or insufficient data. Using search terms for pitolisant (including both generic and brand names such as “pitolisant”, “PITOLISANT HYDROCHLORIDE”, and “WAKIX”), sodium oxybate (including “sodium oxybate”, “LUMRYZ”, “XYREM”, and “XYWAV”), solriamfetol (including “solriamfetol”, “SUNOSI”, and “SOLRIAMFETOL HYDROCHLORIDE”), and modafinil (including “modafinil” and “PROVIGIL”) over the past 5 years were selected through the Food and Drug Administration (FDA, 2024). A total of 101,200 patients were identified after we chose “role_code” as the primary suspected drug.

### 2.2 Data standardization

The related professional terms are standardized according to the ICH International Medical Dictionary for Regular Activities (MedDRA) (Pearson et al., 2009). The system organ class (SOC) is used as the systematic classification standard of ADE, the high light group term (HLGT) describes the subclassification standard of ADE in the same system organ, and the preferred term (PT) is selected as the standard name for a particular ADE. The ADE signal analyses were subsequently conducted for standardization.

### 2.3 Statistical methods

Disproportionality methods were employed to analyze the ADE signals. They are the reporting odds ratio (ROR), proportional reporting ratio (PRR), Bayesian confidence propagation neural network analysis (BCPNN), and multi-item gamma Poisson shrinker (MGPS). The equations and criteria for the four algorithms are set following the standard for signal detection (van Puijenbroek et al., 2002). We acquired a signal when the statistic satisfied the following conditions: lower limit of 95% confidence interval (CI) > 1, a ≥ 3 (ROR); PRR≥2, χ^2^ ≥ 4, a≥3 (PRR); a ≥ 3, IC-2SD > 0 (BCPNN); and lower limit of 95% CI > 2 (MGPS). When a target drug is more likely to be connected to a target ADE than all other drugs are, a higher score will typically be obtained due to a greater disproportionality. All data processing and statistical analyses were performed using Microsoft Excel 2016 and GraphPad Prism 9 (GraphPad Software, CA, United States).

## 3 Results

### 3.1 Demographic data

After deduplicating records with “primary id”, 25,260 patients were included in the final analysis (Table 1). The total number of patients experiencing adverse reactions in the pitolisant group (PG) was 580, while it was 22,473 in the sodium oxybate group (SOG), 1,551 in the solriamfetol group (SG), and 656 in the modafinil group (MG). The majority of the participants were adults, with median ages (in years) of 41(PG), 42 (SOG), 40 (SG) and 43 (MG). In terms of sex, there were 2.4 times more women than men in the PG, 2.6 times more in the SOG, 2.4 more in the SG and 1.6 more in the MG. Consumer, health professional, and physician were the occupations with the most reporting. The United State was the main country that uploaded the cases. When considering the cumulative dose of ADE, the median cumulative doses were 62.3 mg in PG, 27.5 g in SOG, 600 mg in SG, and 73,078.1 mg in MG.

**TABLE 1:** Patient demographic information.

Information	Category	Pitolisant		Sodium oxybate		Solriamfetol		Modafinil	
		Case number	Proportion,%	Case number	Proportion,%	Case number	Proportion,%	Case number	Proportion,%
Age (year)	Median (Q1, Q3)	41 (30, 50)		42 (31, 54)		40 (31,51)		43 (30, 58)	
	[0–18)	7	1.21	336	1.50	10	0.64	17	2.59
	[18–60)	237	40.86	6515	28.99	288	18.57	241	36.74
	[60–100)	28	4.83	1308	5.82	47	3.03	85	12.96
	Unknown	308	53.10	14,314	63.69	1206	77.76	313	47.71
Sex	Female	362	62.41	15,850	70.53	995	64.15	322	49.09
	Male	149	25.69	6059	26.96	409	26.37	203	30.95
	Unknown	69	11.90	564	2.51	147	9.48	131	19.97
Reporter	Consumer	453	78.10	9692	43.13	830	53.51	382	58.23
	Health-professional	49	8.45	5,422	24.13	227	14.64	68	10.37
	Lawyer	0	0.00	2	0.01	0	0.00	0	0.00
	Physician	70	12.07	3,765	16.75	417	26.89	122	18.60
	Other health-professional	1	0.17	254	1.13	7	0.45	25	3.81
	Pharmacist	4	0.69	90	0.40	15	0.97	42	6.40
	Unknown	3	0.52	3,248	14.45	55	3.55	17	2.59
Report country (Top 5)	1	US	95.69	US	97.21	US	90.59	US	67.84
	2	FR	2.41	CA	0.95	FR	3.35	GB	9.60
	3	GB	0.34	FR	0.49	DE	1.74	FR	6.40
	4	IL	0.17	DE	0.22	GB	0.90	CA	4.42
	5	CA	0.17	GB	0.17	CA	0.32	JP	1.98
Report year	2019	7	1.21	4,075	0.18	49	3.16	205	31.25
	2020	106	18.28	3,665	0.16	295	19.02	135	20.58
	2021	79	13.62	4,837	0.22	416	26.82	95	14.48
	2022	327	56.38	3,402	0.15	554	35.72	105	16.01
	2023	61	10.52	6494	0.29	237	15.28	116	17.68
Single dose	Median (Q1, Q3)	9 (8.9, 35.6) (mg)		2.25 (2.25, 3) (g)		75 (75, 150) (mg)		200 (100, 200) (mg)	
Cumulative dose	Median (Q1, Q3)	62.3 (44.5, 64.9) (mg)		27.5 (9, 247.375) (g)		600 (131.25, 3403.125) (mg)		73,078.1 (2272.414, 782664.8) (mg)	

Note: Q1, lower quartile; Q3, upper quartile; US, United States; JP, Japan; IL, Israel; GB, Great Britain; FR, France; DE, Germany; CA, Canada.

### 3.2 Total outcome

There are seven adverse outcomes in the FAERS, ranging from most serious to mild: death, life-threatening, hospitalization-initial or prolonged, disability, congenital anomaly, required intervention to prevent permanent impairment, and others. After removing invalid data, we classified all the outcomes, as shown in Table 2. It seemed that other serious/important medical events ranked first in terms of all the outcomes, followed by hospitalization-initial or prolonged. Significantly, patients with MG exhibited a greater rate of death, while patients with PG and MG demonstrated more pronounced proportions of life-threatening and disabling outcomes than did those with the other two drugs.

**TABLE 2:** Overall outcomes associated with the designated medication.

Outcome	Pitolisant		Sodium oxybate		Solriamfetol		Modafinil	
	Case number	Proportion,%	Case number	Proportion,%	Case number	Proportion,%	Case number	Proportion,%
Death	1	1.33	236	1.33	4	1.52	18	4.96
Life-Threatening	6	8.00	62	0.35	4	1.52	21	5.79
Hospitalization - Initial or Prolonged	22	29.33	3523	19.90	38	14.39	77	21.21
Disability	3	4.00	216	1.22	4	1.52	21	5.79
Congenital Anomaly	0	0.00	3	0.02	1	0.38	22	6.06
Required Intervention to Prevent Permanent Impairment/Damage	0	0.00	3	0.02	1	0.38	0	0.00
Other Serious (Important Medical Event)	43	57.33	13,657	77.16	212	80.30	204	56.20

### 3.3 Comparison of the top 15 ADE signals

**FIGURE 1:**
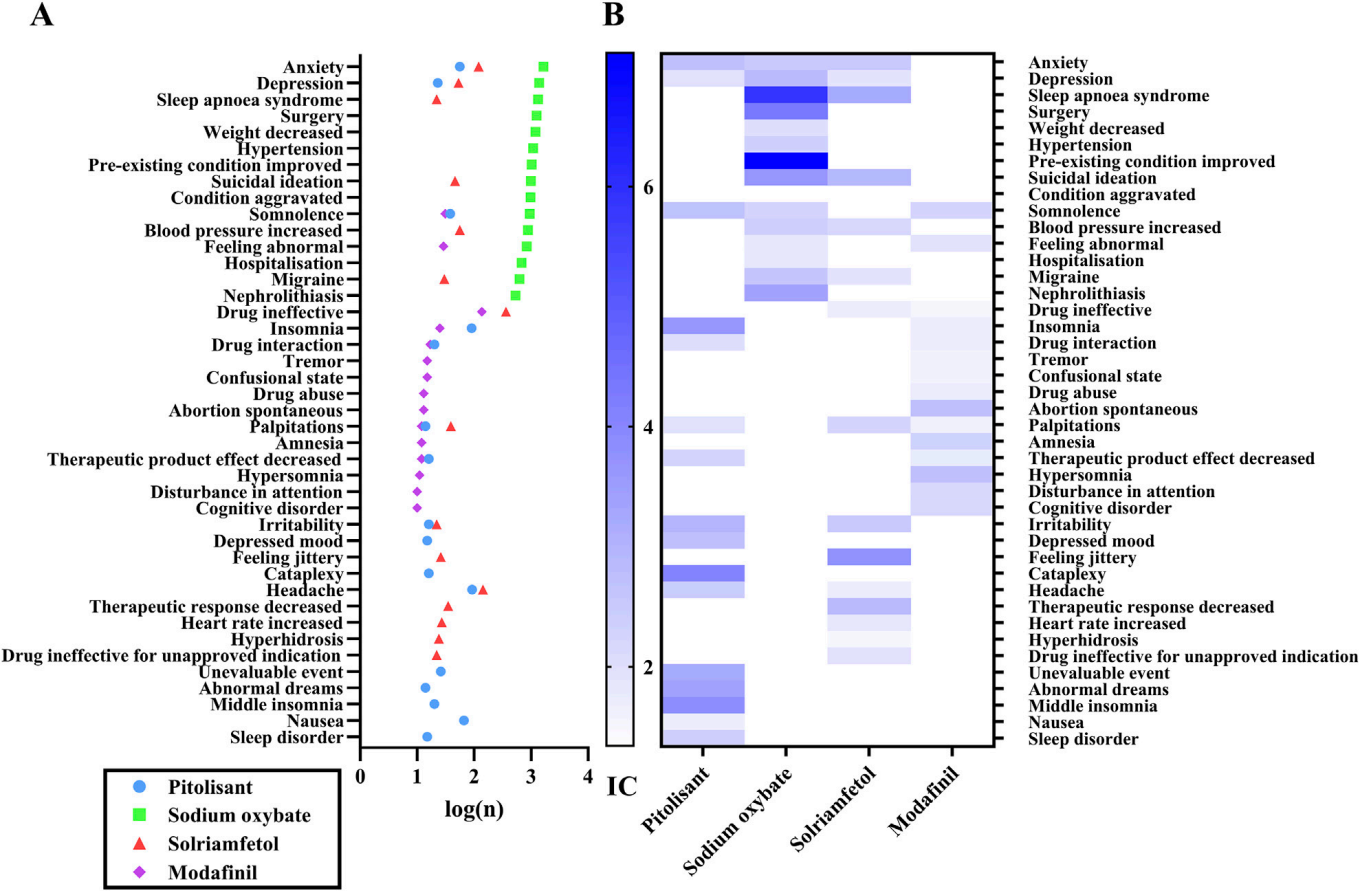
The top fifteen ADEs associated with each medication. Note: 1A, the number of ADEs; 1B, the IC value of ADEs. n, the number of reports; IC, information component, the value in BCPNN method.

The positive ADE signals were analyzed after ROR, PRR, BCPNN and MGPS quantification and threshold setting. A total of 50 signals were recorded, encompassing a cumulative count of 762 cases in the PG, and 640 signals (46,962 cases) in the SOG, 40 signals (1,228 cases) in the SG, and 72 signals (632 cases) in the MG. The top 15 signals according to the number of patients treated with each medicine were compared, as depicted in Figure 1. The number of reports related to anxiety, depression, and sleep apnea syndrome far exceeded those related to other ADEs (Figure 1A). Among them, it is evident that SO contributed the most cases. There are a considerable number of cases associated with drug ineffectiveness in the MG and SG. There are many reports of headache in patients with PG and MG. SG reported greater suicidal ideation. The associations between specific signals and medications were visualized using IC (Figure 1B). Generally, a higher score indicates a stronger correlation, which requires more attention. Sleep apnea syndrome, improved preexisting conditions, suicidal ideation, and nephrolithiasis were prominently observed in the SOG. Both suicidal ideation, sleep apnea syndrome, and improved preexisting conditions were significant in the SG. On the other hand, the PG had higher scores for somnolence, insomnia, and anxiety. Notably, attention was given to several symptoms in the MG group, including spontaneous abortion, hypersomnia, cognitive disorders, and drug abuse.

### 3.4 The proportion of ADE signals at SOC levels

**FIGURE 2:**
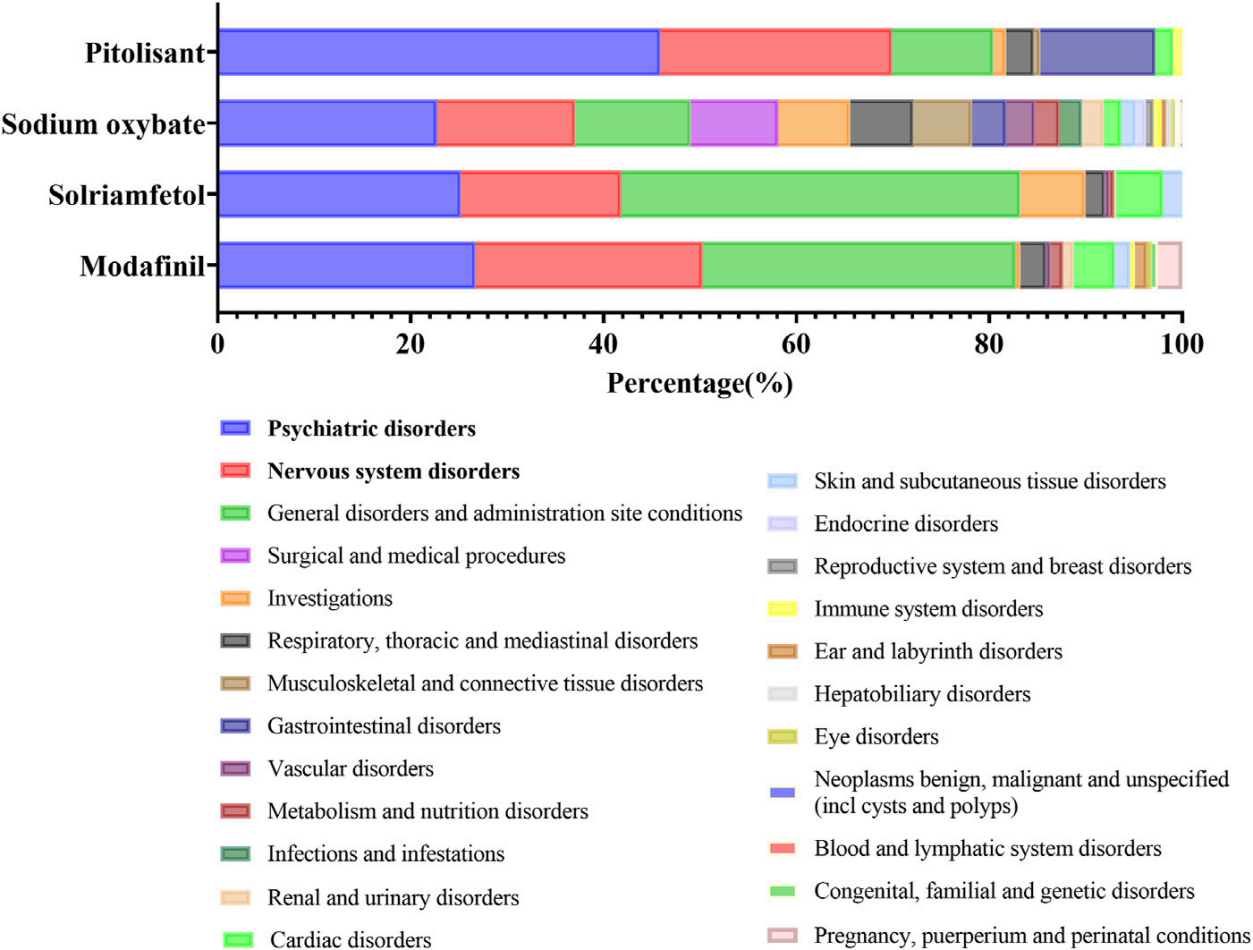
The distribution of ADEs at the SOC level: an overview.

An overview of the proportion of these four medications that resulted in related PTs is depicted in Figure 2. It constituted the predominant majority of psychiatric disorders and nervous system disorders at the SOC level. Moreover, signals pertaining to respiratory, thoracic and mediastinal disorders, as well as cardiac disorders, warrant attention. The prevalence of MG during pregnancy, during puerperium pregnancy and under perinatal conditions is also noteworthy. Subsequently, the potential correlation between an individual ADE in a crucial SOC and signal strength will be specifically discussed at the HLGT level.

### 3.5 ADEs associated with psychiatric disorders at the HLGT level

**FIGURE 3:**
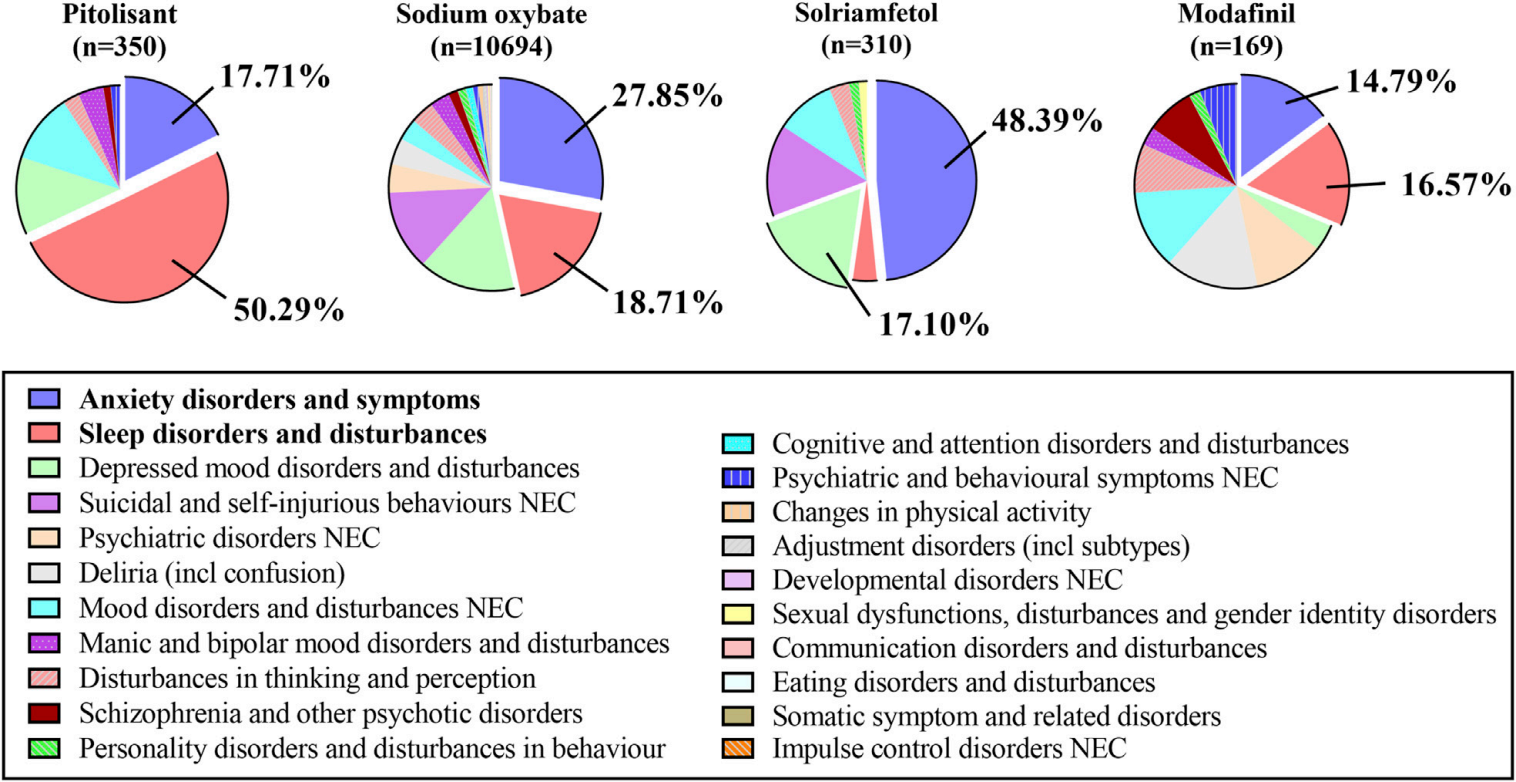
The proportion of ADE signals related to psychiatric disorders at the HLGT level.

The specific analysis of signals related to all psychiatric disorders at the HLGT level revealed that anxiety disorders and symptoms and sleep disorders and disturbances were predominant issues for the PG, SOG, and MG, while anxiety disorders and symptoms, depressive mood disorders and disturbances, and suicide and self-injury and disturbances were major concerns in the SG (see Figure 3). The top five signals were analyzed, as shown in Supplementary Table S1. Even though all the entries in the table are positive signals, we further establish a threshold and categorize them as strong signals with different colors. The four conditions are defined as follows: Condition A (represented by the color blue) indicates that the values of the ROR, PRR, and EBGM exceed 10; Condition B (represented by the color yellow) mandates that the value of the BCPNN exceeds 3; Condition C (represented by the color green) is met when both Condition A and Condition B are concurrently satisfied; and finally, Condition D (highlighted in red) denotes that an individual medicine’s signal strength surpasses that of others. The conditions of insomnia, abnormal dreams, middle insomnia, and initial insomnia were emphasized in green in the PG, signifying their expected significant presence across all four methods. Similarly, enuresis, sleep-related eating disorders, abnormal sleep-related events and parasomnia were all highlighted in green in the SOG dataset.

### 3.6 ADEs related to nervous system disorders at the HLGT level

**FIGURE 4:**
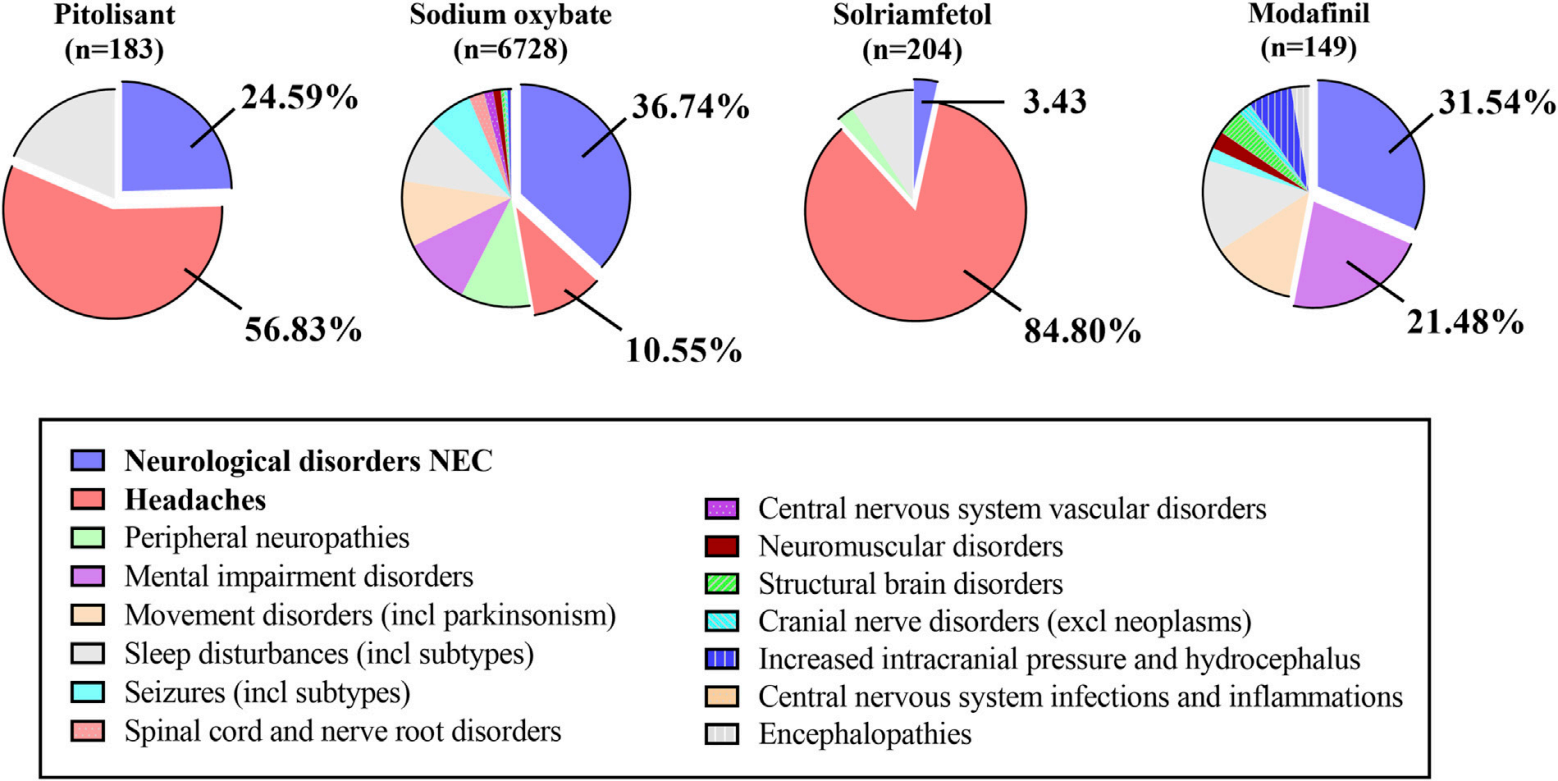
The proportion of ADE signals related to nervous system disorders at the HLGT level.

For nervous system diseases, PG and SOG primarily contribute to the development of the neurological disorders NEC and headaches. SG predominantly presented with headaches. MG mainly resulted in both neurological disorders (NEC) and mental impairment disorders (Figure 4). Similarly, Supplementary Table S2 presents the top five indicators of the nervous system. SOG exhibits a greater number of highly significant signals denoted by green, such as thoracic outlet syndrome, restless legs syndrome, complex regional pain syndrome, and brain fog. Additionally, SOG signals are stronger in individuals with restless leg syndrome, migraine, and carpal tunnel syndrome. Conversely, PG displayed a stronger association with somnolence and headache. Furthermore, the MG exhibited higher scores for nervous system disorders, amnesia, and attention disturbance.

### 3.7 Other significant disorders

PTs in other significant systems, such as respiratory, thoracic and mediastinal disorders; cardiac disorders; and skin and subcutaneous tissue disorders, are discussed (Supplementary Table S3). SOG exhibits pronounced signals in the respiratory system, including upper airway resistance syndrome, central sleep apnea syndrome, exercise-induced asthma, and obstructive sleep apnea syndrome. Moreover, the signal intensity of SOG for sleep apnea syndrome and abnormal respiration is greater than that of SG. SG demonstrated robust signals for sleep apnea syndrome and even stronger signals for tachycardia and palpitations. MG presents numerous strong signals related to skin and subcutaneous tissue disorders and pregnancy, puerperium and perinatal (PPP) conditions. Notably, MG is correlated with spontaneous abortion and fetal growth restriction. Although SOG has a certain impact on PPP-related indicators, it primarily affects maternal factors, whereas MG may be associated with adverse fetal outcomes.

## 4 Discussion

The occurrence of narcolepsy may be influenced by various factors, including polygenic susceptibility, autoimmune factors, and infections (Han et al., 2012; Partinen et al., 2014; Mahoney et al., 2019). Both genders can be affected by narcolepsy. According to the European Narcolepsy Network, there is no significant sex difference among narcolepsy patients (Khatami et al., 2016). However, a retrospective study conducted in China revealed a male-to-female ratio of 2:1 among narcolepsy patients with cataplexy (Wu et al., 2014). Nevertheless, this study demonstrated a significantly greater proportion of ADEs in females than in males (Table 1). These findings emphasize the importance of heightened vigilance for potential ADEs when administering treatment to female patients.

There was a diagnostic delay observed in narcolepsy, as indicated by the European study which reported a mean age of onset for EDS in the patient cohort of 20.9 ± 11.8 years with a standard deviation, while the mean age at diagnosis was 30.5 ± 14.9 years (Zhang et al., 2022). Although narcolepsy can manifest in children under the age of 10, symptoms typically reach their peak during the second decade of life, with a primary peak occurring during adolescence and possibly a secondary peak around the age of 30–39 (Dauvilliers et al., 2001; Kornum et al., 2017). Our study revealed that ADEs associated with these medications usually occur at a median age of approximately 40 years, which closely aligns with the second peak. ADEs were most common among adults aged 18–60 years, followed by the elderly population (over 60 years old), while teenagers accounted for the lowest proportion. The occurrence of ADEs in adolescents and elderly individuals can be attributed to the early onset of the disease and its potential lifelong consequences.

The primary objective of most symptomatic treatments should be to optimize the sleep-wake cycle in patients with narcolepsy, with a specific emphasis on enhancing daytime performance. Enhancing EDS and mitigating cataplexy are typically of utmost significance. In general, medications that increase norepinephrine or dopamine release or inhibit their reuptake have wake-promoting effects and are useful in managing EDS. Conversely, medications that inhibit serotonin or norepinephrine reuptake have anti-cataplectic effects (Thorpy, 2020). Modulation of GABAB receptors or H3 receptors has effects on both EDS and cataplexy. The most approved treatments for EDS associated with narcolepsy include pitolisant, SO, solriamfetol, and modafinil (Syed, 2016; Powell et al., 2020; Bassetti et al., 2021).

Pitolisant has demonstrated minimal abuse potential in both preclinical and clinical studies, making it the only noncontrolled substance among anti-narcoleptic medications in the United States (Lamb, 2020). Clinical trials have shown that pitolisant effectively improves EDS and is well tolerated compared to modafinil (Dauvilliers et al., 2013). It has also exhibited efficacy in alleviating narcolepsy symptoms in children, with a safety profile similar to that observed in adults; however, further research is required to confirm its long-term safety (Dauvilliers et al., 2023). The most commonly reported adverse effects among both adults and children were headache, insomnia, and anxiety (Lamb, 2020; Dauvilliers et al., 2023). The findings of this study align with the aforementioned conclusions.

The prescription of sodium oxybate (SO) is restricted due to its classification as a central nervous system depressant (Robinson and Keating, 2007). Almost 10.3% of patients treated with SO discontinued treatment because of adverse reactions (FDA, 2024). It should be emphasized that the use of SO provides a black warning concerning the possible development of respiratory depression. After conducting further analysis of ADEs related to the respiratory system, we identified several noteworthy findings. Specifically, sleep apnea syndrome (with 1326 cases, ROR = 80.77, PRR = 76.06, BCPNN = 5.89, EBGM = 62.18), respiratory depression (n = 104, ROR = 8.80, PRR = 8.76, BCPNN = 3.00, EBGM = 8.56), apnea (n = 95, ROR = 15.84, PRR = 15.77, BCPNN = 3.72, EBGM = 15.11), and upper airway resistance syndrome (n = 14, ROR = 1565.58, PRR = 1563.61, BCPNN = 3.83, EBGM = 276.75) were found to be significant concerns in our analysis. In the past, respiratory arrest and death have been reported in cases of severe SO intoxication (Mason and Kerns, 2002). Recently, medication-induced central sleep apnea has emerged as one of the eight recognized categories contributing to this condition (Javaheri et al., 2024). Both opioid and non-opioid medications, including SO, can trigger this phenomenon, which generally resolves upon discontinuation of the offending agents. Therefore, it is crucial to recognize these ADEs.

Additionally, clinical cases have reported psychosis and suicide attempts as secondary effects of SO (Ortega-Albas et al., 2010; Chien et al., 2013). In our study, a significantly high proportion of adverse reactions to SO were attributed to abnormalities in the psychiatric system (as depicted in Figure 2), particularly anxiety, depression, and suicidal ideation. Among these, depression and suicidal ideation exhibited the highest number of cases and strongest correlation intensity compared to the others (refer to Supplementary Table S1). The impact of GABA on blood pressure has been a subject of ongoing discourse (Persson and Henning, 1980). However, certain scholars argue that the administration of SO does not pose any additional cardiovascular risks for patients with narcolepsy (Avidan and Kushida, 2020). Nevertheless, we observed a significant association between SO and conditions related to blood pressure, including hypertension (n = 1081, ROR = 5.36, PRR = 5.15, BCPNN = 2.34, EBGM = 5.09), gestational hypertension (n = 10, ROR = 8.30, PRR = 8.29, BCPNN = 2.30, EBGM = 8.12), and preeclampsia (n = 21, ROR = 4.81, PRR = 4.81, BCPNN = 2.02, EBGM = 4.75). Furthermore, it is worth noting the presence of SO signals in pregnancy and congenital familial diseases.

Solriamfetol is classified as a federally controlled substance. The safety and tolerability of solriamfetol have consistently been demonstrated in clinical studies, with commonly reported adverse reactions typically occurring within the initial 2 weeks of treatment and mostly resolving within that timeframe (Hoy, 2023). Research has indicated that the therapeutic efficacy of solriamfetol for managing EDS in patients with narcolepsy or obstructive sleep apnea remains unaffected by a history of depression (Krystal et al., 2022). Among adults with solriamfetol, the most frequently observed adverse reactions include headache, decreased appetite, nausea, anxiety, and insomnia (Winter et al., 2023). The findings of our research are largely consistent with those of previous reports, although we identified several noteworthy new adverse reactions, including drug inefficacy, suicidal ideation, restless legs syndrome, and somnambulism, with a high degree of association strength. Close monitoring of changes in heart rate is imperative during the administration of this medication due to the highest proportion of reports and strongest correlation found in tachycardia and palpitations. A randomized controlled trial investigating the effects of solriamfetol on QTcF intervals in healthy participants demonstrated that neither dose of solriamfetol resulted in a QTcF prolongation exceeding 10 milliseconds, with the most frequently reported ADEs being nausea, dizziness, and palpitations (Zomorodi et al., 2021).

The available studies on modafinil in pregnant women are insufficient and lack proper control, resulting in its classification as pregnancy category C. The administration of modafinil in pregnant women remains a subject of controversy. Some studies have demonstrated a potential increased risk of major congenital malformations following exposure to modafinil during pregnancy (Damkier and Broe, 2020; Kaplan et al., 2021). However, one study indicated that the use of modafinil during early pregnancy was not significantly associated with an elevated risk of major malformations (Cesta et al., 2020). In our analysis, we observed a connection between modafinil and restricted fetal growth, spontaneous abortion, and cognitive disorders. In addition, given the substantial number of reports and positive signals, maintaining vigilance toward drug inefficacy and abuse is crucial.

## 5 Conclusion

A meticulous analysis of pharmacovigilance data on pitolisant, sodium oxybate, solriamfetol, and modafinil in real-world settings has contributed to the identification of potential novel or notable ADE signals. The majority of observed adverse reactions in this study were consistent with those listed in the product instructions. However, based on an analysis of demographic characteristics, we recommend enhanced monitoring for female patients with narcolepsy when utilizing these medications due to their greater proportion of ADEs. Given that these drugs primarily target the central nervous system, prioritizing the safety of psychiatric and neurological medication is crucial. This study provides valuable insights into the selection of appropriate drugs by comparing the distribution and signal intensity of ADEs across different organ systems. It is anticipated that this paper will offer additional information regarding safe and rational medication for narcolepsy—a rare and disabling condition.

## 6 Limitations

Our research has several limitations. Due to the voluntary reporting nature of these reactions from an uncertain population size, establishing a causal relationship with drug exposure or reliably estimating their frequency is not always feasible. First, ADE cases in the FAERS database are reported spontaneously, leading to bias due to incomplete or missing information. Second, it is challenging to control for confounding factors such as dose, comorbidities, and drug combinations despite selecting drugs with a “primary suspect” designation. Third, usage rate data could not be obtained from the database; thus, morbidity information could not be provided. Our primary focus was on determining the proportion of related ADEs and assessing the strength of the association between medication and target ADE signals. Subsequent utilization of these results would necessitate epidemiological studies and clinical trials.

## Data Availability

The raw data supporting the conclusions of this article will be made available by the authors, without undue reservation.
